# How Can the Lived Environment Support Healthy Ageing? A Spatial Indicators Framework for the Assessment of Age-Friendly Communities

**DOI:** 10.3390/ijerph17207685

**Published:** 2020-10-21

**Authors:** Melanie Davern, Rachel Winterton, Kathleen Brasher, Geoff Woolcock

**Affiliations:** 1 Healthy Liveable Cities Group, Centre for Urban Research, RMIT University, Melbourne, VIC 3000, Australia; 2 Centre for Health Equity, Melbourne School of Population and Global Health, University of Melbourne, Parkville, VIC 3010, Australia; 3 John Richards Centre for Rural Ageing Research, La Trobe Rural Health School, La Trobe University, Bendigo, VIC 3550, Australia; R.Winterton@latrobe.edu.au; 4 Age-Friendly Northeast Victoria, Central Hume Primary Care Partnership, Benalla, VIC 3672, Australia; Kathleen.Brasher@centralhumepcp.org; 5 Institute for Resilient Regions, University of Southern Queensland, Toowoomba, QLD 4350, Australia; Geoffrey.Woolcock@usq.edu.au

**Keywords:** age-friendly, health, indicators, planning, tools, spatial, neighbourhoods

## Abstract

The Age-Friendly Cities and Communities Guide was released by the World Health Organization over a decade ago with the aim of creating environments that support healthy ageing. The comprehensive framework includes the domains of outdoor spaces and buildings, transportation, housing, social participation, respect and inclusion, civic participation and employment, communication and information, and community and health services. A major critique of the age-friendly community movement has argued for a more clearly defined scope of actions, the need to measure or quantify results and increase the connections to policy and funding levers. This paper provides a quantifiable spatial indicators framework to assess local lived environments according to each Age-Friendly Cities and Communities (AFC) domain. The selection of these AFC spatial indicators can be applied within local neighbourhoods, census tracts, suburbs, municipalities, or cities with minimal resource requirements other than applied spatial analysis, which addresses past critiques of the Age-Friendly Community movement. The framework has great potential for applications within local, national, and international policy and planning contexts in the future.

## 1. Introduction

Research has long recognized that environmental factors play a significant role in determining health and wellbeing in older age [1], and there are rising proportions of older people in the populations across the world. Consequently, the recently released United Nations Decade of Healthy Ageing 2020–2030 calls for sustained global action to generate transformative change in four priority areas: addressing ageism; creating age-friendly communities; delivering integrated and person centered care; and providing long-term care [2,3].

Increased urbanization and policy discourses supporting ageing in place add to the urgency to create and plan for age-friendly environments. On a global scale, life expectancy has increased from 47 years in the mid-20th century to an expected 78 years by the mid-21st century [4] and 21% of the world’s population is predicted to be aged over 60 years by 2050 [5]. The World Health Organization (WHO) World Report on Ageing and Health [6] documented how age-friendly environments play a significant role in preventing or delaying many of the health changes related to biological ageing and chronic disease. Age-friendly environments can support people to continue to live as independently as possible in their local communities and allow ageing to take place in the social contexts of social and built environments [2]. 

The social and environmental determinants of healthy ageing are reflected in the eight domains of the WHO Global Age-Friendly Cities and Communities guide [7]. While some local adaption of these domains occur, they are designed as fit for purpose [8] for place-based actions in content areas that are important to later life [9]. Since its inception in 2007, the Age-Friendly Cities and Communities (AFC) movement has evolved into a global movement, with urban and rural communities undertaking action to improve the quality of life for older people in each of these eight domains. Very importantly, AFC supports long term action for ageing in place, which is needed to plan and build cities and towns of the future.

Previous critiques of the AFC movement argued for a narrower scope of actions that are better quantified and connected to policy levers [10]. These suggestions are well justified. Although the movement has brought much attention to planning for ageing, and has been informed by the voice of older people themselves, there has been little: (i) uptake on the community scale [11]; (ii) recognition of AFC in peer-reviewed publications [12]; or (iii) direct application in urban planning [13]. Consequently, this prevents AFC actions being adopted or aligned within existing planning processes, particularly at the municipal level where much AFC policy and planning needs to occur. 

In more recent years, the ability to measure age-friendliness across diverse international contexts has also become an important consideration within the age-friendly movement [8,14]. In 2015, the WHO Kobe Centre developed [15] a set of core indicators for age-friendliness to support cities that are part of the WHO Global Network of Age-Friendly Cities and Communities [1]. The WHO report proposed measures relating to wellbeing, equity, and accessible physical and inclusive social environments. In the same year, the Public Health Agency of Canada [16] released an evaluation guide comprising thirty-nine indicators directly relevant to the eight AFC domains. The indicators were selected to provide community-level measures for both quantitative and qualitative outcomes with four indicators to measure longer-term health and social outcomes [17].

In 2018, the WHO Regional Office for Europe synthesized the existing international approach into seven sets of tools to measure and communicate the assessment and monitoring of age-friendly initiatives in Europe [8]. However, there is concern that many of the suggested indicators and measures for the assessment and monitoring of AFC are subjective in nature [14,17] or survey based [12]. These existing approaches are expensive to administer at the community level and beyond the budgets of many local governments who ultimately have the responsibility for local area planning. They are also often tailored to local environments, limiting the ability of state or national governments to have comparative assessments of age-friendly initiatives. New resources are needed to support practitioners and planners seeking to assess, monitor, and promote age-friendliness in the local environments where people live to support healthy ageing in place.

### Age-Friendly Communities and the Lived Environment

Age-friendly communities comprise three distinct and interrelated constructs: local liveability; meeting the needs of older people; and ageing as a lifespan developmental process [18]. A set of validated liveability indicators that promote health and wellbeing have been developed for general populations [19]. These provide specific aspects of urban liveability associated with positive health outcomes, including transport [20], walkability [21], public open space [22], housing [23], employment [24], social infrastructure [25], and food environments [26]. 

This paper builds on these liveability indicators to propose objective spatial indicators to assess the age-friendliness of lived environments. The term “lived environment” is adopted throughout this paper in preference to a built environment to reflect this broader assessment of the key features needed to support healthy ageing. This extends beyond the common conception of the built environment, which often includes transportation systems, land development patterns, and microscale urban design (e.g., footpaths) [27,28]. A lived environment reflects the importance of locality and access to good urban design, as well as human-made and natural environments to support health and wellbeing in the local neighbourhoods where people live. This is consistent with the argument regarding the narrow application of the term ”built environment” where both human made and natural worlds are conceived as though there is no separation between them [29].

Spatial indicators provide a quantitative measurement of local lived environments using geocoded data (defined by x and y co-ordinates) developed using Geographic Information Systems (GIS). Data linked to a street address can be mapped using GIS and calculated as spatial indicators, providing aggregated measures across a range of geographic areas, including neighbourhoods or census tracts, suburbs, municipalities, regions, or states. Aggregated geocoded data can be drawn from a range of existing administrative data sources that assess the lived environment and a range of social, economic, and environmental issues. Spatial AFC indicators consequently provide objective and cost-effective assessments of age-friendliness that are easily replicated across large geographic areas using desktop spatial analysis. These indicators can also be made readily accessible to local governments using online digital planning portals and liveability indicator systems for cities, like the Australian Urban Observatory (auo.org.au) [30]. 

The development of quantifiable spatial indicators of AFC addresses the major critiques of the AFC initiative—that it is too descriptive in approach [31], not measured or monitored by indicators [31], and without a clear understanding of an indicator framework [32]. This paper proposes spatial indicator tools that can be applied for the assessment of AFC in local lived environments using a GIS methodology. 

These AFC spatial indicators can also be applied in a variety of international contexts with direct relevance to the Healthy Cities movement [33], the New Urban Agenda, and the 2030 Agenda for Sustainable Development [34]. The 2030 Agenda provides a global framework for sustainable urban development up until 2030 signed by all 193 members states with 169 specific targets. These include Sustainable Development Goals (SDGs) with specific mention of older people in targets for Goal 10 Reduced Inequalities, Goal 11 Sustainable Cities and Communities, and Goal 17 Partnerships for the Goals. In addition, the Decade of Healthy Ageing [3] calls for disaggregated data in twenty-eight indicators across eleven Goals. 

Spatial indicators measuring AFC in lived environments are noted by the United Nations as being necessary for the measurement and monitoring of any actions contributing to sustainable development (Goal 17) and multi-stakeholder partnership development and policy and institutional coherence. They have been developed to address segregation or siloed approaches in the current planning approaches and to encourage discussion and action that can promote integrated policy, planning, and practice across urban planning and public health. Often the outcome of AFC remains the sole responsibility of health or social planning with little integration across important portfolios, such as transport or statutory or strategic planning. The implementation of AFC principles must extend beyond practitioners with interest in ageing and should ideally be integrated across policy portfolios with budget and legislative support. 

This paper aims to introduce a new set of AFC spatial indicators that can be used to quantify and assess the age-friendliness of local lived environments and monitor changes in age-friendliness over time consistent with the SDGs and 2030 Agenda. These indicators seek to support the Decade of Healthy Ageing, which includes a commitment to action in the development of age-friendly environments and improved measurement, monitoring, and research [8] as well as tools to support planners and practitioners working within government settings. These spatial indicators of AFC also identify the importance of older people and their lived environments in sustainable urban development and the 2030 Agenda.

## 2. Materials and Methods

**Figure 1:**
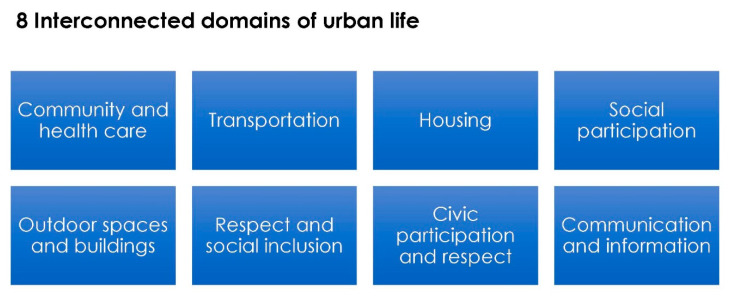
The eight interconnected domains of the Age-Friendly Communities framework [35].

Eight interconnected domains are included in AFC (Figure 1). The selection of specific spatial indicators to assess the lived environment of each AFC domain was made following a workshop held with all five authors to identify the most relevant measures for each of these domains. The multidisciplinary experience of the research team spans gerontology, public health, urban planning, psychology, epidemiology, sociology, health geography, health policy, governance, and community development.

Potential indicators were then judged against the key criteria recommended by the WHO (Box 1) as well as other best practice principles for indicator application [36] including: direct links to policy; connection to theory and existing research; available time series data; connection to budgeting and planning; relevance to most people; and connection to lived reality.

These latter criteria being understood and relevant to most people, particularly older people, are particularly important and informed by previous research in the development of a specific indicator of access to services for older people [37], which included focus groups of older people to determine the local needs and services of highest importance. The selected measures also needed to be relevant to the majority of older people living in a wide range of lived environments, and to measure the most critical requirements for places that support AFC principles.

**Box 1. The criteria suggested for defining local AFC indicators [15].**

**Measurable:** Will variations in the indicator be observable over time due to specific actions?**Disaggregation possible:** Can the indicator be disaggregated by gender, age group, or across neighbourhoods? There are also other strategies that could be important in the local context, including ethnicity, socioeconomic status, etc.**Aligns with local goals and targets:** Does the indicator link to a broader local agenda?**Can be linked to action:** Does the indicator provide an understanding of the various actions that might need to be undertaken?**Within local influence:** Does the local government or community have the mandate or authority to act on this indicator? For example, a federal insurance scheme is mostly beyond the influence of the municipal government.**Easy to collect:** Are the data required to produce the indicator easy to collect in a timely manner?**Socially acceptable:** Is the collection of this information acceptable to the communities and individuals concerned?

## 3. Results

The following section describes each of the selected AFC spatial indicators with research evidence provided to support each indicator (Table 1). 

### Selection of Measures

The suggested spatial indicators for each AFC domain are presented in Table 1 with the priority indicators notated with asterisks. This provides flexibility for practitioners in identifying the key spatial indicators of importance to AFC or additional optional indicators where resources are available. Additional information is provided below explaining why these indicators are recommended for each AFC domain with detailed explanations of the supporting research evidence. 

The indicators recommended in the following section were identified in accordance with indicators acting as icebergs and highlighting issues of major importance [37]. Only after the major factors have been quantitively assessed should further qualitative assessment be completed, similar to a hierarchy of need. For example, if there are no public open spaces available there is little point in assessing the maintenance, shelter, or facilities available in public open spaces within an area. Additional qualitative assessment could also include local consultation with older residents and relevant stakeholders.

## 4. Outdoor Spaces and Buildings

The priority indicators identified for this domain are walkability for transport [38,39] and access to public open space within 400 m [22]. These indicators are directly related to walking [40,41,42], specifically in older people [43], and associated with physical health benefits [44] and mental health benefits [45]. 

Walkable neighbourhoods are important for older people because, along with the fact that they enable people to reach destinations with commercial and social opportunities [43,46], walking is also associated with maintaining functional independence [47] and better cognitive function [48]. Similarly, public open spaces that are easy to visit with walkable access are important for older people and important in reducing social isolation and increasing physical activity [49].

**Figure 2:**
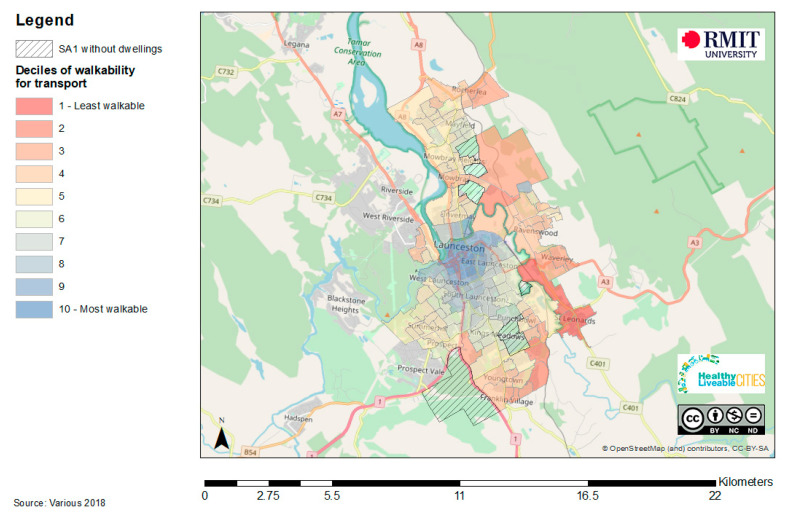
An example of a walkability assessment for the city of Launceston, Tasmania, Australia [52].

Data required to create indicators of walkability are commonly available within municipal and planning contexts. Road network analysis (a way to walk), land use mix (destinations to walk to), and housing density (people to service the destinations) are common key components of walkability assessments. Similarly, public open space location data are also regularly held by most municipal governments. Footpaths are an important infrastructure supporting walking in older people [50,51], and walkability can also be refined by superimposing footpath access where spatial data are available. An example of a walkability for transport assessment for the regional city of Launceston in Tasmania, Australia was calculated and is provided in Figure 2 to demonstrate the value of neighbourhood level walkability assessments. The results clearly suggest that the inner neighbourhoods of the city of Launceston have good walkability while the outer neighbourhoods are less supportive of walking for transport, particularly those on the eastern side of town.

Additional spatial indicators for consideration include intersections with visual and auditory signalled pedestrian crossings that allow time for older people to cross over roads, and particularly busy intersections [53,54]. In Australia, many regional towns avoid the use of signalized pedestrian crossings and opt for roundabout intersections, which encourage continual traffic flow and can be frightening for people with reduced mobility. 

Access to public seating is also recommended to be available within local public open spaces to encourage rest stops while walking (overlapping with the suggested measure of accessibility to public open space). Clean and safe public toilets are also recommended, including those with accessibility features [51] and should also be included within high quality public open spaces. Accessible buildings are italicised in Table 1 due to the difficulty in sourcing data that measure buildings developed according to universal design principles. If possible, these are recommended, as older people experience difficulties associated with access to public buildings and the lack of handrails, narrow corridors, and steps [51]. Post Occupancy Evaluations are generally more common in sustainability assessments [55] and are time and staff resource intensive but could be considered as an alternative measure if no other data are available to assess buildings.

There is a growing body of evidence showing a positive association between healthy ageing and blue space [56]. This is worthy of future consideration but is not accessible within all lived environments and, hence, has not been included as a recommended measure within the outdoor space and building domain but could be considered as second tier measures. Blue space is defined as outdoor environments (natural or manmade) that prominently feature water and are accessible proximally (being located in, on, or near water) or distally/virtually (being able to see, hear, or sense water) [57]. Therapeutic design of a built environment using urban green and blue infrastructure was shown to be protective for healthy ageing while supporting those with cognitive decline, or illness [58]. 

Similarly, a study of largely older people in Hong Kong found that general health was significantly higher in people with a sea view from their home [59], while, in Ireland, older people had a lower risk of depression in those with more sea views [60]. In addition, nature-based solutions, through green and blue space urban management planning, can mitigate the health impacts of climate change while addressing the need for climate resilience in local communities [61]. Future revisions of the AFC principles could consider the inclusion of more detailed measures of green and blue spaces in the domain of outdoor spaces and buildings to address changing climates around the globe. These could include access to local blue spaces, public and private tree canopy coverage, public street tree canopy coverage and the associated shade capability, in combination with the currently recommended measures of walkability and accessibility to public open space. These measures are very worthy of consideration but bring their own challenges in terms of data access and spatial capability making them harder to produce. Consequently, they are suggested as potential expanded, not essential, measures of the AFC lived environment assessment. 

## 5. Transport

Transport is an important determinant of health [62,63] influencing access to local services, engagement in paid and non-paid productive activities (such as employment or volunteering), maintaining and developing social networks and supports, and engaging in social and recreational activities. Public transport has also been identified as a critical influence of liveability in a community [19] and active transport important to older people [64]. Policy-relevant spatial public transport indicators are typically based on 400 m access or a 5-min walk [20,65]. Another important factor that influences the use of public transport is service frequency. Consequently, access to any public transport stop provides a high-level assessment while access to frequent public transport provides a more refined assessment. Similar measures are also included in the Australian Government’s National Cities Performance Framework (https://www.bitre.gov.au/national-cities-performance-framework).

For older people, mobility is essential for social participation and wellbeing [66]. Public transport is particularly important for older people who might have a reduced ability to drive. Older people tend to use public transport more frequently if there is easy access to public transport in neighbourhoods at a distance less than 5 min away [67]. This is also consistent with existing research that found that the frequency of public transport and wait time affected older people’s willingness to travel [68] and that a high proportion of older people are no longer driving [69]. 

Data for these indicators can most often be sourced from public access data portals, Open Street Map or General Transit Feed Specification (GTFS) where public transport data are provided by transport agencies into a computer readable format for web developers [70]. Gaining access to more detailed data describing public transport that meets Disability Standards is another very valid indicator and has been associated with increased satisfaction and perceived useability in older people [71]. Similarly, access to a bus stop with an accompanying shelter and seat is also important for older people’s mobility, as well as dropped curves, footpaths, and pedestrian signals [54].

## 6. Housing

Housing is central to living a productive, meaningful, and healthy life, and housing quality is an important influence on self-reported health [72]. Unaffordable housing is detrimental to mental health in low to moderate income households [73]. Unaffordable housing has also been associated with an increased risk of poor self-rated health, hypertension, and arthritis, and renting, rather than owing a home, increases associations between unaffordable housing and self-rated health [74]. Consequently, housing costs and gentrification [75] are particularly important to consider, with housing stress in lower income households being a particularly important indicator for the assessment of age-friendly cities. 

Housing needs, sizes, and types can change as people age. Older people might consider downsizing to smaller homes with reduced maintenance needs or to be closer to extended family for support to age in place [76]. In rural and regional areas, older people might need to move from larger farms and back into towns where services are more readily available. Alternatively, frail older residents might require the support of aged care providers to support high care needs. Addressing these issues means that communities need to understand the available housing diversity options (e.g., larger houses, smaller houses, units, and apartments to serve broad community needs) as well as access to services for residents. 

AFC supports multiple housing options that are beneficial to all residents with many municipalities thinking primarily about formal aged-care accommodation when addressing housing needs for older people. Even more concerning in Australia, it is common for aged care facilities to be built on the outskirts of cities and towns where there is an abundance of inexpensive and undeveloped land. This isolates older people from the rest of the community, makes it harder for people to access and visit, decreases access to other community services, and decreases intergenerational contact within communities.

The 30/40 housing affordability indicator is recommended and describes the proportion of households in the bottom 40% of household incomes spending more than 30% of their income on housing costs [77]. This measure is also referred to as the Ontario measure where the interest in housing affordability first identified the disproportionate impact of housing costs on lower income households [78,79]. Understanding community demographic profiles, particularly age, in combination with the high incidence of 30/40 housing affordability issues should raise concerns for any community wanting to support age-friendliness. Specifically, older adults on an aged pension within the private rental market will face significant challenges in housing affordability [80]. 

The indicator of access to services for older people was developed with older people themselves [37] and includes hospitals, General Practitioners, Aged Care Facilities, public transport stops, supermarkets, community centres, libraries, and Universities of the 3rd Age, and could also include places of worship and parks. This indicator also provides a useful assessment for the AFC domain of Community Support and Health Services but is included in the housing domain to reinforce the importance of urban planning that supports the co-locations of services and housing options. The proportion of government owned dwellings could also be investigated as an additional support measure of AFC, particularly in lower income areas. 

## 7. Social Participation

Meaningful social relationships and participation are essential for good health, with health defined as a social phenomenon in the social determinants of health [81]. Social participation has been associated with physical activity [82], mental health [83], reduced psychological distress [84], reduced risk of myocardial infarction [85], and up to a 50% increased likelihood of survival in people with strong social relationships compared to lifestyle risk factors [86]. 

For older people, social participation provides greater life satisfaction [87], is protective against cognitive decline [88], and contributes to resilience in older people [89], especially in rural communities [90]. Social participation is also being taken seriously internationally, and the United Kingdom appointed a new Minister for Loneliness and a national government action plan on loneliness [91]. 

The recommended spatial indicators supporting social participation connect to the access to services for older people [92] that are included in the housing domain. Two indicators are recommended: access to community centres and neighbourhood houses; and access to recreational services that cater to the needs of older people. Shared or ‘third spaces’ such as these are critical social infrastructure [25] and essential in supporting social participation for older adults [93]. Recreational services also support physical and mental health through opportunities for physical activity designed for older people and supporting community connections. 

Another indicator recommended for inclusion is access to a local library, which also supports the AFC domains of respect and social isolation, communications and information, and community support and health services. Libraries provide multiple community benefits beyond simply lending books [94,95], including multimedia borrowing, technology training, community classes, lectures, and opportunities for intergenerational and community connections. Libraries also support the need for learning opportunities across the course of life with Universities of the Third Age (U3As) providing social and learning benefits to older people [96,97]. This is associated with better physical health and activity levels [98]. Places of worship are also considered an important facilitator of social connections and social capital [99], particularly in humanitarian arrivals [100] and different cultures [101,102].

## 8. Respect and Social Inclusion 

Respect and social inclusion are essential to ensure social participation for older people. There is much debate on the definition of social inclusion, though most studies refer to an objective participation in society and a more subjective assessment of whether the actual participation meets an individual’s preferences [103]. Most definitions of social exclusion emphasise the importance of social activities as a core component [104]. However, the effects of cumulative disadvantage, decreasing social networks, and age discrimination magnify the negative health and wellbeing impacts of social exclusion in later life [105].

A local or lived environment must provide accessible buildings, housing and transport, along with opportunities for social activities to occur if social inclusion and social participation are supported and encouraged. Previous research on the services deemed important for older people has emphasised the importance of local services, such as shops [37,69], and this is supported by the use of new spatial indicators that can access formal and informal places to meet. These include recommended indicators of access to social clubs/senior citizens clubs or participation in international clubs, like Rotary or Probus, that are more formally organised by older people themselves. Alternatively, informal opportunities for social inclusion include an indicator of distance-based access to local cafes that support broader intergenerational social opportunities. Older people need a range of venues to create opportunities for social activities as a foundation for community respect and social inclusion. 

## 9. Civic Participation and Employment

Empowerment, autonomy and control [63,106], and employment conditions [107] were all found to be important influences of actual and self-reported health. Control over one’s own destiny has also been proposed [106], consistent with an understanding of health being simultaneously influenced at the individual (micro/personal), place and community context (meso/community) as well as the larger societal context (macro/societal level) [108]. 

Civic participation and employment are important influences of agency and autonomy in a society. Consequently, it is important to understand how many older people are engaged in paid and unpaid productive activity in the community. This is best measured through the proportion of people who remain employed past the official retirement age (66 years in Australia noting there is no official retirement age and eligibility for the aged pension is currently 66 years increasing to 67 years by 2023) or people aged 60 years or more who are engaged in regular volunteering. These indicators of paid and unpaid productive activity are also important measures of social engagement and civic participation and could be separated into additional age brackets or deciles (e.g., 60–75 years) for more detailed information. It is important to note that employment is also not defined according to hours worked, acknowledging both the civic connections and benefits that come from any level of paid employment and that retirement is not a single event and includes a diverse range of retirement patterns [109]. 

There has been criticism regarding the dominance of volunteering in measures of collective civic social participation in older people [110] with voting participation argued as a better measure of civic participation [111]. However, voting participation is less relevant in countries like Australia where electoral voting is compulsory and volunteering activities are measured every 4 years. Volunteering is also particularly important in regional areas of Australia where third sector or non-profit organisations rely on older people volunteering [112] with increasing proportions of older people residing in rural locations [113]. In countries where voting is not compulsory (e.g., the USA), then voting participation could be considered as an additional measure of civic engagement.

## 10. Communications and Information

In 2016, approximately 86% of Australian households had access to the internet [114]. This proportion decreased to 77% in remote areas where it is common to have a high proportion of older people within populations, with entertainment, social networking, and banking the most commonly supported activities supported by internet connection.

Internet access is also becoming more necessary to access information about the government, health, banking, and community services as well as to maintain contact with friends and family. Finding information on services like these is also critical for older people to age in place and is necessary to support independent living and the connection to communities [115]. Th information provision also extends beyond essential services and includes services provided by local libraries, which includes online books, audio, audio-visual, and educational resources that can be made available online for people with physical or geographical mobility restrictions. Online streaming (e.g., Netflix) is another more recent example of recreational activities supporting social connection and information provision. However, all these online resources require household internet access. 

Access to a national radio service is another important source of information and becomes particularly important in emergency management, including preparation and recovery from natural disasters, such as floods, droughts, and bushfires, which are becoming increasingly more commonplace in Australia. Emergency SMS messaging systems are also deployed during emergency situations to inform residents of impending safety risks but are worthless without adequate mobile phone reception. Climate change is predicted to increase the likelihood of these emergency situations making telecommunications assessment essential in the support of AFC. It is also necessary for developing technologies, including passive surveillance of movement monitoring within the home, personal alarm devices, and telehealth [116], which have become increasingly accessible and necessary during the 2020 Coronaviruses 2019 (COVID-19) pandemic. 

Communication is an important influence on the wellbeing of older people [117], and both household internet and mobile phone reception provide essential telecommunication systems that support both intergenerational communication with family and friends, the communication of essential information [118], and the ongoing adoption of new technologies [119], as well as influence the quality of life [120]. Currently, there is a paucity of references or inclusion of technological solutions offered to support AFC and healthy ageing and technology, and ICTs have recently been suggested as a new smart age-friendly ecosystem framework [118]. Suggestions included in this new framework to assist AFC include: the development of smart housing; the inclusion of ageing in smart cities and engagement with the Internet of Things (IoT); the better use of digital assistants (e.g., Alexa) in the home; the use of digital robots for deliveries; electronic camera enabled doorbells; and motion sensors to detect mobility. Technological features like these require inclusion during new housing development and have benefits across multiple AFC domains beyond communication. They also require a rethink and interdisciplinary collaboration between planners, architects, developers, computer science, industry, and the government. While the opportunities are waiting for action, they also require engagement with older people themselves and their families using qualitative and ethnographic research methods [121]. This is an important area of growth and future development in AFC and requires further research. 

## 11. Community Support and Health Services

Access to primary health support services is essential and necessary for people to age in place. It is also the preferred option for most older people to maximise their health and wellbeing [122]. Within the local community, access to General Practitioners has been identified by older people themselves as essential community support services [36,69,123] and the key access point for primary health care. Consequently, access to General Practitioners was identified as an indicator of primary importance within community support and health services. These practitioners also provide gateway services and referrals to any other medical specialists, including geriatricians, who specialise in treating conditions that affect older people, including dementia. 

Additional indicators that should be included relate to housing support either as in-home support packages or residential aged-care accommodation. All of these services are also included within a complete definition of social infrastructure, which has an important influence on subjective wellbeing [25] and are important components of liveability [19]. 

### A Regional Case Study Example

The approaches and spatial measures described above were applied in a case study in a regional context and rural centre in north-eastern Victoria, Australia. The regional town is located over 200 km north-east of the capital city of Melbourne in the centre of the state of Victoria, south-eastern Australia. The major industries are agriculture and manufacturing, with a population of over 9000 people. Both the state government department of health and the local municipality/council were interested in analysing and understanding AFC and broader liveability given an increasingly ageing rural population. 

The spatial measures used to assess this included: walkability (with and without footpaths); access to public open space; access to public transport; housing affordability; housing diversity; government owned dwellings (social housing); access to services for older people; libraries; universities of the 3rd Age; places of worship; volunteering; households with internet access; aged care facilities; and access to General Practitioners. The results were presented to the local health department officials, the local municipality, and as a community presentation to residents at the local library. 

Many of the challenges and barriers to AFC planning were identified in the spatial measures and were confirmed by the lived experiences of residents from the local community. These included: poor walkability on the outer areas of town; difficulty getting to doctors and medical services located at the regional hospital located on the outer town boundary with limited public transport and poor walkability; disconnection between the older people, families, and younger people in the town due to the location of residential aged care on the town boundary next to the hospital; the importance of cafes and social spaces in the centre of town to support community and social connections; the value of the town’s library, art facilities, and public open spaces; and inequity in the disadvantaged areas of the town that had reduced access to public transport and lower levels of household internet connections. The use of mapped spatial measures of AFC was hugely beneficial for inter-agency conversations and planning initiatives as well as community conversations, engagement, and validation of the spatial analyses. The results also highlight the future negative impact of the age-friendliness of the town if future residential aged care development is supported in the outer areas of the town. 

## 12. Discussion

The original WHO Global Age-Friendly Cities Guide was developed in response to the rapid population ageing and urbanisation across the world and was informed by interviews conducted with older people themselves in over 33 different countries [7,15]. The ultimate aim of AFC is to create environments that support healthy ageing. This paper provided detailed, objective, and functional spatial measures of age-friendliness across lived environments that can be used to assess, monitor, evaluate, and communicate age-friendliness refined to the neighbourhood level. Objective spatial measures of the lived environment are critical for the following reasons: to simplify assessments of AFC; to provide a foundation level of knowledge about the age-friendliness of an environment; to assist local and state government planning by informing and monitoring future actions and interventions needed to promote healthy ageing in communities; and to include older people into targets of the 2030 Sustainable Development Goals and the New Urban Agenda. 

The movement has previously been criticised for a lack of objective measurements and the need to connect these ideals into functional measures connected to policy, planning, and financial levers [10]. Previous attempts at developing indicators of age-friendliness have been non-specific, non-coordinated, and reliant on survey-based responses (e.g., World Health Organization [15]). Such assessments are also beyond the budget, resources, capabilities, and motivation of local planning agencies and municipalities. The proposed spatial measures of age-friendliness across lived environments is relevant to planners, policymakers, advocacy organisations, governments, architects, industry, citizens and research audiences. The suggested indicators are provided to guide and inform discussions and interventions to promote healthy ageing. The measures can also be adopted and customised to local environments ranging in geographic and population sizes, rurality, climate conditions, and resource limitations.

The proposed spatial indicators of AFC address these issues through the application of GIS technology to produce an objective assessment of the age-friendliness of local lived environments, drawing on indicators from the liveability literature that are specifically relevant to the values, preferences, and needs of older adults. These indicators provide measurement and quantification of AFC domains consistent with the idea that value comes with measurement and leads to knowledge production as argued by Lord Kelvin over 200 hundred years ago [124]. The more simplistic interpretation of this, is that what is measured, is valued, and consequently is done. 

### 12.1. Linking Spatial AFC Indicators to Policy Contexts Outside of Ageing

One of the critical issues raised in the recommended AFC spatial indicators is the connection of all indicators within existing policy and planning contexts [13]. All the recommended indicators can be linked to existing policy and planning environments regardless of whether these have a local/municipal, state, or national focus. The connection of indicators to policy has been long identified within social indicator research [125,126]. 

These indicators can assist governments in meeting their commitments to the Sustainable Development Goals in a way that is meaningful for a growing segment of their populations. There is also an increasing interest and development in public health digital observatories. For example, relevant liveability indicators for the 21 largest cities of Australia are available in the Australian Urban Observatory (auo.org.au) launched in 2020. There is an opportunity to make spatial indicators available through novel data visualisation and ease of communication providing an influence on the policies required for healthy ageing across communities. 

The spatial indicators recommended for assessing AFC domains can all be influenced and improved through policy levers. This includes the indicators suggested for outdoor spaces, transport, housing, social participation, respect and social inclusion, civic participation and employment, communications and information, community support, and health services. The indicator results can be influenced though local and immediate strategies or applied in advocacy with the responsible higher government agencies. This can include reviewing AFC assessments within the context of current policy contexts, existing public health planning, liveability planning, transport planning, strategic planning, land use, and statutory planning 

It is also important to acknowledge the limitations of AFC spatial indicators and understanding that these aggregated area-based results effectively act as icebergs of knowledge [35] providing a tip of the iceberg assessment of what is occurring, with additional information required to understand why the result is happening and how it can be addressed. Consequently, the objective AFC spatial indicators should also be combined with additional sources of knowledge. These include consultation and engagement with local older people themselves to expand understanding, prioritise actions, and support the greatest social and economic benefits and returns on investments that support improved health and quality of life for older people. 

### 12.2. Unique Contexts, Regional and Rural Localities 

Given the diversity of cities, communities, and places, it is recognised that the achievement of all suggested indicators might not be feasible across all geographic settings. This is particularly relevant to rural and regional locales, which often have a lower population density and reduced levels of physical or social infrastructure. Consultations with older people and combining subjective understandings with more objective AFC spatial indicators will also help to inform the understanding of unique community contexts, including regional and remote areas. 

For example, high levels of walkability might not be possible across an entire town in a rural area with a small population. Alternatively, signaled pedestrian crossings might not be necessary. However, a walkability assessment using the recommended walkability indicator could identify walking and transport barriers (e.g., a major road or bridge across a rail line) or identify the best location for new community services. Alternatively, the distances and measures of accessibility listed within indicators may vary across diverse rural and regional settings, but as noted above, these definitions of access within indicators must be determined through consultation with the older adults and communities to a reach consensus on what can be reasonably expected within this locale. 

Consequently, in certain settings, these proposed indicators should act as a tool to prompt place-specific discussions around what is important in terms of measurement indicators, and what is achievable (particularly in relation to what should constitute reasonable access). A notable challenge of AFC planning is the absence of the relevant climate change implications in the current AFC principles and domains and inclusion of ICT and new technology. We recommend that future revision of AFC should expand and account for the challenges associated with climate change given the implications on the health and wellbeing of older people [127] and the ultimate AFC goal of healthy ageing. The relationship between older people’s physical health and mental health with the environment, urban design, architecture, and AFC could also be considered in the development of future indicators [128].

## 13. Conclusions

Understanding and expanding AFC spatial indicators for unique contexts and environments is needed in the future and this current foundation of recommended indicators can be applied and tested across a range of different locations. This could include localities with climatic extremes (e.g., heat, cold, and snow), regional and rural locations, international comparisons, and cultural differences to explore how communities differ and what additional indicators should be included. The major aim of this research was to propose a foundational set of objective AFC spatial indicators that can be applied in any location with minimal resources and are directly aligned for policy intervention. This is particularly relevant to planning and policymakers working in government and was neither previously available nor consistently applied within AFC locations. Further research should investigate how this proposed suite of AFC spatial indicators can be added to, refined, or customized to address the needs of many different locations, including the relevant subjective indicators to enhance knowledge. The inclusion of new technology and ICT and addressing climate change are also increasing areas of interest in the future.

## Figures and Tables

**Table 1:** Age-Friendly Communities (AFC) domains and suggested spatial indicators.

AFC Domains	Suggested Spatial Indicators for AFC Assessment and Monitoring
1. Outdoor spaces and buildings	Walkability for transport (with and without footpaths) * Access to public open space within 400 m * Intersections serviced with pedestrian crossings Access to public seating Access to public toilets (with and without accessibility features) *Accessible buildings*
2. Transport	Access to a public transport stop within 400 m * Access to a public transport stop within 400 m with a regular service every 30 min (7 a.m.–7 p.m.) * Access to public transport with Disability Standards for Accessible Public Transport Bus stops with seats/shelters Disabled car parking access Community transport measure (if possible)
3. Housing	Proportion of households in the bottom 40% of incomes spending more than 30% of income on housing costs * Housing diversity according to eight different housing types Proportion of government owned dwellings Access to services for older people [37] *
4. Social Participation	Access to neighbourhood houses/community centres * Recreational services catered to older people e.g., a YMCA * Access to libraries Access to Universities of the 3rd Age (U3As) Access to places of worship
5. Respect and social inclusion	Access to social clubs/senior citizens clubs * Access to local cafés measured by distance * Membership of Clubs like Probus and Rotary
6. Civic participation and employment	Proportion of population aged 60+ years regularly volunteering * Proportion of population working beyond official retirement age (currently 66 years in Australia) *
7. Communications and information	Proportion of households with access to the internet * Proportion of households with mobile phone reception Access to ABC or national broadcaster radio
8. Community support and health services	Access to General Practitioners * Access to Geriatricians Access to residential aged care accommodation Access to Commonwealth Support Home Packages (funding supporting ageing in the home if available) *
Additional contextual factors for consideration include: the Estimated Resident Population; proportion of population aged more than 60 years; population age distribution including proportions of older and younger populations in area; ethnicity; education; homeownership; residential density; remoteness e.g., Accessibility/Remoteness Indices or the distance between towns in rural settings; the risk of natural disasters; climatic conditions; and the impact of climate change.	

* Recommended as priority indicators for inclusion.
